# Towards a General Theory of Neural Computation Based on Prediction by Single Neurons

**DOI:** 10.1371/journal.pone.0003298

**Published:** 2008-10-01

**Authors:** Christopher D. Fiorillo

**Affiliations:** Department of Neurobiology, Stanford University, Stanford, California, United States of America; Indiana University, United States of America

## Abstract

Although there has been tremendous progress in understanding the mechanics of the nervous system, there has not been a general theory of its computational function. Here I present a theory that relates the established biophysical properties of single generic neurons to principles of Bayesian probability theory, reinforcement learning and efficient coding. I suggest that this theory addresses the general computational problem facing the nervous system. Each neuron is proposed to mirror the function of the whole system in learning to predict aspects of the world related to future reward. According to the model, a typical neuron receives current information about the state of the world from a subset of its excitatory synaptic inputs, and prior information from its other inputs. Prior information would be contributed by synaptic inputs representing distinct regions of space, and by different types of non-synaptic, voltage-regulated channels representing distinct periods of the past. The neuron's membrane voltage is proposed to signal the difference between current and prior information (“prediction error” or “surprise”). A neuron would apply a Hebbian plasticity rule to select those excitatory inputs that are the most closely correlated with reward but are the least predictable, since unpredictable inputs provide the neuron with the most “new” information about future reward. To minimize the error in its predictions and to respond only when excitation is “new and surprising,” the neuron selects amongst its prior information sources through an anti-Hebbian rule. The unique inputs of a mature neuron would therefore result from learning about spatial and temporal patterns in its local environment, and by extension, the external world. Thus the theory describes how the structure of the mature nervous system could reflect the structure of the external world, and how the complexity and intelligence of the system might develop from a population of undifferentiated neurons, each implementing similar learning algorithms.

## Introduction

Our knowledge of the computational function of the nervous system remains limited and no general theory has emerged. Perhaps the most obvious difficulty in developing a computational theory is the complexity of the system, with its large and diverse population of neurons, each with its own unique connectivity. However, we know that the entire system develops from a single cell, and thus it may be possible to identify relatively simple principles that shape the structure and function of all neurons. The present work proposes that each neuron shares the same basic computational function, and that function mirrors that of the system as a whole.

In analogy to the conceptual framework suggested by Marr [1], a general computational theory of the nervous system should contribute to our understanding at three distinct levels of analysis. First, a general theory would need to identify a single computational goal that is broad enough to cover the entire nervous system and to subsume all of the more specific computational problems that the nervous system encounters. Second, the theory should describe how the computational goal is achieved; that is, how information is organized in the nervous system and how it flows through space and time. Finally, the theory should specify the physical mechanisms that implement the computation, the molecular and cellular processes that hold and transform information. The present theory attempts to address all three of these levels.

Central to the theory is the proposition that all neurons operate according to shared computational principles. Below I outline the theory in the form of five hypotheses. These address the general computational goal of the system (1), the organization of information in a single neuron (2), the rules that govern the selection of a neuron's inputs, or information sources (3 and 4), and the organization of the system (5). Whereas much past work has focused on describing our information about the nervous system, I suggest here a fundamentally distinct approach in which the goal is to characterize the information the nervous system possesses about its world. This approach, outlined below under hypothesis 1, is made possible by combining Bayesian probability theory with biophysics.

## Results

### Hypothesis 1: The Computational Goal

*The computational goal of the nervous system is to minimize uncertainty (maximize information) about the state of the world (or more specifically, an aspect of the world that could be referred to as “future reward”).*

From a biological perspective, the goal of all nervous systems is to select motor outputs in order to promote the future of an animal's genetic information. It is proposed that the only problem in making such decisions is uncertainty about the state of the world. If the system could accurately predict the state of the world, then the problem would be solved, and the system would merely select the output that it knows will maximize its expected future reward. The process of minimizing uncertainty is thus considered to be formally identical to the process of “decision-making,” since decisions are rendered trivial in the absence of uncertainty. The system does not need to concern itself with the state of the world in general, but only with a part of the world that I will refer to as “future reward” (defined below under hypothesis 4). The computational goal of the system described here is similar to that found in the field of reinforcement learning, [e.g. 2]–[4]. However, although the brain is specifically concerned with future reward, this goal is otherwise equivalent to the proposal that the brain must use its limited information to predict or estimate the state of the world, an idea that dates back at least to the work of von Helmholz [5] and which remains prominent today [e.g. 6], [7].

The proposal described above is perhaps already the dominant view of the computational problem facing the brain. It is widely agreed that the nervous system is an information processing system, and information is defined solely by its ability to reduce uncertainty. However, I suggest here that there has been confusion surrounding the concept of information, and I propose what I believe to be a novel, strictly Bayesian approach to the biophysical information of neurons. This particular approach to information is critical to the claim that the present theory addresses the fundamental computational goal of minimizing uncertainty (or maximizing information) about the world.

A prediction necessarily involves uncertainty, and it is therefore properly described in terms of a probability distribution of potential states of the world. Uncertainty refers to the width of the probability distribution (as quantified by the distribution's entropy), and it is inversely related to information. The greater the information, the narrower the probability distribution and the lower the uncertainty. Information and uncertainty cannot be specified mathematically without first determining a probability distribution. Although probability theory has been widely used to describe neural function, contradictory definitions of probability have been proposed, and there are different approaches that can be taken in applying the concept of probability to the nervous system (Text S1). The present work applies a strictly “Bayesian” definition to probabilities, as described by Jaynes [8] (as opposed to a “frequentist” definition, which equates probabilities with frequencies). According to a Bayesian view, probabilities are always conditional on a set of information. There are rules of logic that relate a set of information to a probability distribution. For example, the principle of maximum entropy requires that we fully acknowledge our ignorance by considering all possibilities equally probable unless we have evidence to the contrary. Thus if the only information available is that an event has four possible outcomes, then the probability of each is 0.25 (since the probabilities must sum to one and the flat distribution is the one with maximum entropy).

A Bayesian understanding of probability provides us with two equally valid but very distinct approaches to describing neural function. We can either describe our information about a nervous system and its environment, or we can describe a nervous system's information about its environment. Whereas the former “third-person” perspective has often been utilized [e.g. 6], I suggest here that the latter “first-person” perspective may provide for a simpler and more compact description of neural function. To this end, I describe below how we can take “the neuron's point of view” by determining a probability distribution of potential states of the external world conditional only on information held within the biophysical structure of the neuron. This approach is distinct from previous work, which derived probability distributions that were not conditional on information known to be found within a neuron [e.g. 6]. The novelty of my approach arises from the definition of probability, rather than from any distinct interpretation of biophysics. For a more detailed discussion of the different approaches to quantifying a neuron's information, see Text S1.

**Figure 1:**
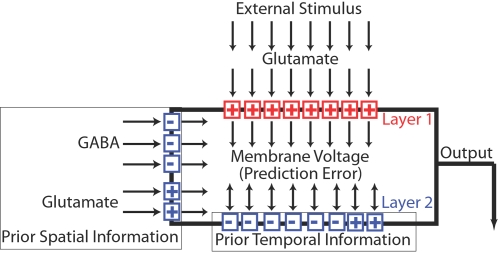
Schematic illustration of a model neuron. Arrows indicate the direction of information flow. A typical neuron receives inputs from the sensory periphery via glutamate, which depolarizes the membrane potential (“+”). The glutamate-gated ion channels and synapses that mediate this response are referred to as layer 1. They define the neuron's stimulus (the “excitatory center” of its receptive field). The function of layer 1 is to provide current information about the external world. Those individual inputs that are most successful in depolarizing the neuron, and which are most closely correlated with reward, are selected according to a Hebbian or error-maximizing rule (equation 4). The neuron's other ion channels constitute layer 2. The function of layer 2 is to use prior information to predict membrane voltage, and thereby predict the conductance of layer 1 and glutamate concentration as well. The membrane voltage is determined by the difference between the output of layer 1 and its expected output as determined by layer 2 (equation 1), and it therefore functions as a prediction error. In predicting voltage, layer 2 acts to drive voltage towards a point near the middle of its range where the error is zero. The ion channels of layer 2 are selected to perform this function by an anti-Hebbian or error-minimizing rule (equation 3). Many of these ion channels are inhibitory (“−”) and tend to open when the neuron is depolarized, whereas others are excitatory (“+”) and tend to open when the neuron is hyperpolarized. Some are gated by membrane voltage and provide prior temporal information, whereas others are gated by neurotransmitters and contribute prior spatial information.

A neuron's information must be about something, and thus we must first define the “subject” of a neuron's information (what it is that a neuron is predicting). Each neuron possesses information about some aspect of the world that I will define as the neuron's “stimulus.” Although the word “stimulus” is often associated with concrete sensory aspects of the world, I use it here in a broader sense that would also apply to the much more abstract subject matter of the information in a high-level cortical or motor neuron. If a neuron is close to the sensory periphery, then it may be relatively straightforward for us to precisely specify its stimulus. For example, a photoreceptor possesses information about the intensity of light of particular wavelengths in a particular region of space. The stimulus of a neuron further from the sensory periphery is more abstract, and as a practical matter it may be difficult for us to specify precisely. However, although each neuron is presumed to possess information about some aspect of the external world (broadly conceived), a neuron must also possess information about its local environment. The proximal surrogate of a neuron's external stimulus is proposed to be the local concentration of a neurotransmitter summed across a set of individual synapses (Fig. 1). For most neurons this would be an excitatory neurotransmitter such as glutamate. A typical neuron is envisioned as being linked to the sensory periphery through a feed-forward series of excitatory neurons. Thus, by possessing information about local glutamate concentration, a neuron would also possess information about its external stimulus.

**Figure 2:**
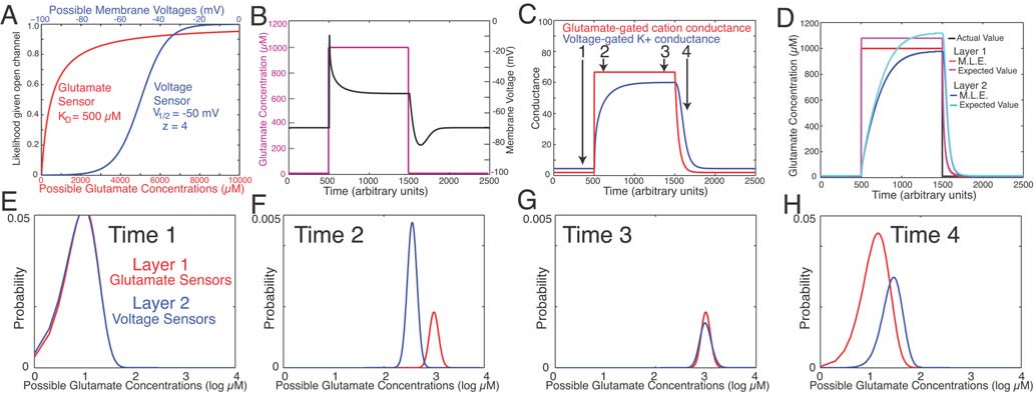
Estimates of glutamate concentration by glutamate-gated cation channels (“layer 1”) and by voltage-gated K+ channels (“layer 2”) in a model neuron that has 100 channels of each type. See Methods and Text S1 for details. A. Estimates made by single two-state sensors in their *on* conformations (equations 5–7). The glutamate sensor (red) had an equilibrium dissociation constant (KD) of 500 µM. The voltage sensor (blue) had 4 elementary charges (z), and the voltage at which either state was equally likely (V_1/2_) was −50 mV. B. Glutamate concentration (magenta) was stepped from 10 to 1000 µM, which evoked a membrane depolarization that declined with time (black). C. The conductance of glutamate-gated cation channels and voltage-gated K+ channels. In each case the maximal possible conductance was 100. D. Maximum likelihood estimates and expected values of glutamate concentration conditional only on information present in the populations of sensors in layers 1 and 2. E–H. Probability distributions of glutamate concentrations at time points 1–4, as indicated in panel C. Each of these distributions is entirely conditional on the information of layer 1 or layer 2. Note that glutamate concentration is presented on a logarithmic scale, and that the y-axes differ in F and G relative to E and H.

If a neuron possesses information about the intensity of its stimulus, then we can say that it estimates or predicts its stimulus (“estimate” and “predict” are used here as synonyms, and “prediction” could apply to the present as well as the future). To quantify a neuron's prediction, we would like to find the probability distribution of possible stimulus intensities conditional exclusively on the information possessed by the neuron. A neuron gathers information about its stimulus through sensors (Fig. S1), such as rhodopsin or glutamate receptors, which are coupled to ion channels and thereby influence the neuron's membrane voltage. As described in Methods, the Maxwell-Boltzmann equation of statistical mechanics (equation 5) specifies the likelihood of various stimulus intensities given the state of a sensor (Fig. 2). We can therefore determine the probability distribution of potential stimulus intensities conditional only on the information in one or more sensors (Fig. 2). Thus, merely by deploying sensors in its plasma membrane, the neuron performs the critical function of predicting stimulus intensity. The prediction is necessarily accompanied by a reduction in uncertainty (relative to the complete uncertainty and flat distribution that would accompany the absence of sensors), and in principle, the reduction in uncertainty can be precisely quantified.

The simple two-state sensor described in the Maxwell-Boltzmann equation is assumed to be the fundamental substrate of information. The entire theory is concerned with the arrangement of sensors within the nervous system, since this arrangement naturally determines the flow of information. The sensors that are of primary concern here are those found in ion channels. However, I use the term “sensor” because it is a general term and it has a simple relationship to the Maxwell-Boltzmann equation. Indeed, protein molecules such as ion channels usually consist of multiple two-state sensors. Sensors can be arranged in parallel or in series. If a population of sensors all directly sense the same stimulus, such as the sensors found in the rhodopsin molecules of a photoreceptor cell, then those sensors are in parallel to one another and it is useful to refer to the entire population as a single “layer.” Within a layer, each sensor contributes additional information to the layer's estimate of stimulus intensity. Sensors can also be arranged in series, so that information can be communicated from “upstream” to “downstream” sensors. A downstream sensor may be found in the same protein molecule, in a distinct molecule in the same neuron, or in a downstream neuron. An example, described below and in figure 2, is that of a first layer of glutamate-gated channels and a second layer of voltage-gated potassium channels. A second layer of sensors would “sense” and thereby estimate the output or state of the first layer. In doing so, the second layer would be indirectly estimating the stimulus or input to the first layer. In an idealized (though unrealistic) case, the second layer would possess a perfect copy of the information in the first layer, although it would necessarily receive that information after the first layer. Because of the communication made possible by a series of sensors, a sensor of membrane voltage could contain information about glutamate concentration (Fig. 2), and at the systems level, the sensors in a cortical neuron could contain information about a quantity external to the nervous system. In Text S1, I describe in greater detail the principles by which we can determine probability distributions of stimulus intensities conditional only on the information contained in multiple sensors, arranged either in parallel or in series.

### Hypothesis 2: Prediction Error

*A neuron is proposed to integrate current information about its stimulus from one pool of ion channels and synapses, and prior information from another pool. Its membrane potential signals prediction error (the difference between its current and prior information).*

The simplest scenario to imagine would be that a neuron estimates the intensity of its stimulus (as described above) and communicates that estimate to downstream neurons. However, it is known that neurons preferentially signal changes in stimulus intensity. We also know that a neuron's output is influenced by many types of ion channels that do not directly sense the neuron's stimulus. Many of these ion channels are proposed to contribute prior information about the stimulus. In the present model a neuron has an expectation of stimulus intensity based on prior information, and it only produces a positive output signal when stimulus intensity exceeds its expectation. Thus a neuron can be said to signal “prediction error.”

By using its membrane potential to signal prediction errors, the neuron is efficient in only teaching itself what it does not already know, and in only telling downstream neurons what they have not already been told. Thus prediction errors promote efficient communication [e.g. 9]–[13]. They are also used to drive plasticity in learning algorithms, where they allow a system to identify the internal and external events that are the earliest and best predictors of a stimulus [e.g. 2]–[4], [14]. Observation of animal behavior suggests that learning in the nervous system is driven by prediction errors [15]. Although error signals have previously been described within the nervous system [2], [3], [9]–[13], [16], the present work suggests that single generic neurons are inherently designed to signal prediction errors.

The present model of a neuron is illustrated schematically in figure 1. A neuron's membrane voltage (or output) depends on two functional layers of sensor-gated ion channels. Layer 1 directly senses current stimulus intensity, whereas layer 2 receives prior information about the same stimulus indirectly (from other points in space and time). I propose further below that the ion channels and synapses of layer one may be selected by a Hebbian rule (hypothesis 4), whereas those of layer 2 may be selected by an anti-Hebbian rule (hypothesis 3). To illustrate the prediction of stimulus intensity by layers 1 and 2, we can consider a simplified neuron with just two types of ion channel. Although I focus here on a graded-potential, single-compartment neuron, the same principles are proposed to apply to spiking neurons and to the computational function of dendritic compartments and pre-synaptic terminals (Text S1). The neuron's first layer consists of glutamate-gated channels that are permeable to the cations sodium and potassium. Binding of glutamate therefore depolarizes the membrane voltage towards the cation equilibrium potential (E_cat_∼0 mV). The neuron's second layer consists of voltage-gated potassium channels (E_K_∼−100 mV). Membrane voltage approaches a steady-state value (*V*_∞_) of
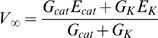
(1)where *G_ca_*_t_ and *G_K_* are the cation and K+ conductances. The response of such a neuron to a square-wave pulse of glutamate is shown in figure 2B–C. Information about glutamate concentration naturally gets to glutamate-gated channels before it gests to K+ channels. The estimate of glutamate concentration by the K+ channels therefore lags behind the estimate made by the glutamate receptors (Fig. 2E–H). Thus the voltage-gated K+ channels can be said to use prior temporal information. Which period of the past the K+ channels use to predict the present depends on their kinetic properties (Text S1). When the estimate of glutamate concentration by the first layer (glutamate-gated channels) exceeds the estimate made by the second layer (voltage-gated K+ channels), the neuron is depolarized (Fig. 2). The neuron is hyperpolarized when the opposite is true. Thus the membrane voltage can be thought of as a prediction error.

The goal of the neuron is to accurately predict its stimulus, which means minimizing its error. If membrane voltage corresponds to the error, then ion channels contributing prior information (layer 2) should modulate their activity in order to drive voltage towards the middle of its range where the error is zero (Fig. 2). Thus K+ and Cl- channels should tend to be open when stimulus intensity is high and the neuron would otherwise be depolarized, whereas depolarizing channels of layer 2 should tend to be open when the neuron would otherwise be hyperpolarized. Depolarizing channels of layer 2 would include non-selective cation channels distinct from those of layer 1 (this could include glutamate-gated channels at a distinct subset of synapses), but possibly also including channels selective for sodium or calcium. However, some sodium and calcium channels are presumed to serve distinct roles in long-distance communication and plasticity, respectively (Text S1), and they could therefore be entirely outside of both layers 1 and 2. The prevalence of K+ channels activated by depolarization, and non-selective cation channels opened by hyperpolarization, is consistent with the present hypothesis, since these channels would usually tend to stabilize membrane potential. In addition, it has been found that inhibitory conductances in cortical and tectal neurons tend to be activated at the same time as stimulus-driven excitatory conductances, thus canceling or “predicting away” the excitation [17]–[20]. Some of the best evidence for this hypothesis comes from studies of the retina.

Neurons rely on spatial and temporal correlations to predict light intensity as accurately and as early as possible. There are strong positive correlations between light intensities at neighboring points in space and time, and there is substantial evidence that neurons in the retina exploit these correlations to predict their stimulus (light intensity in the receptive field center) [e.g. 9], [10], [13], [21]. These predictions are evident in such familiar phenomena as light adaptation and surround inhibition. Adaptation results primarily from prior information about stimulus intensity carried through time by molecules that are intrinsic to a neuron, such as voltage-activated K+ channels. Spatial prior information would be carried through neural circuitry and would activate neurotransmitter-gated channels. The prototypical example would be GABA-gated chloride and potassium channels. (Note that relative to information from the excitatory center, information from the inhibitory surround would typically be delayed by communication through an additional neuron, and its prediction is thus from the preceding moment in time.) Spatial information refers to space in the nervous system, which does not necessarily correspond to external space. Thus it would include information derived from correlations between colors and between tones, in addition to correlations through external space, since all of these are represented by discrete neurons at the sensory periphery.

The description of a neuron's output as signaling prediction error is proposed to be useful for understanding the organization and flow of information in neurons, and to emphasize that the goal of the neuron is to predict the state of its stimulus. However, the concept could be seen as a useful means of describing what we already know about neurons, rather than as a new hypothesis about neuronal function. The signaling of prediction errors does not in itself significantly constrain the relationship between a neuron's inputs and outputs. This is because a neuron's output depends on its prior information (the prediction made by layer 2), but we generally do not know what prior information a neuron has, and the notion of prediction error does not necessarily tell us anything about a neuron's prior information. Below I discuss the rules by which a neuron may select amongst its sources of prior information.

### Hypothesis 3: Selection of Prior Information Sources

*A neuron's sources of prior information (including GABA synapses and different types of voltage-dependent K+ channels) are selected to minimize its prediction error. One way this could occur is through an anti-Hebbian type plasticity rule.*

By merely deploying sensors, a neuron reduces its uncertainty about the intensity of its stimulus. Sensors devoted to any period of the past and any region of space would be informative. However, some would be more informative than others, resulting in smaller prediction errors and less uncertainty. It is proposed that a neuron should select its prior information sources in order to efficiently minimize its errors. This could be done by regulating the number of functional ion channels of a particular type or within particular synapses. The goal of the following discussion is to delineate the rules that determine a neuron's inputs. Neither the mechanisms nor the timescales of plasticity are a fundamental concern here. Thus the proposed principles of selection could be implemented in the adult system, during development, or exclusively through natural selection over generations. Although the emphasis here is on activity dependent plasticity rules that could be implemented within the lifetime of a single neuron, the more critical point to the general theory concerns the “solutions” towards which the system converges, even if this occurs only through natural selection.

The inputs that contribute prior information span a spectrum of points in space and time, and a neuron is proposed to select those inputs that best minimize the error in predicting stimulus intensity. Which period of the past a channel represents depends on its kinetic properties (Text S1). There are numerous types of non-synaptic ion channels that differ in their kinetic properties as well as in their voltage dependence, with the diversity of potassium channels being particularly striking [22]. A mature neuron expresses only a subset of these ion channels. It is proposed that the pattern of a neuron's stimulus, acting via the voltage-mediated error signal, would select the types of non-synaptic ion channel, and the corresponding periods of the past, that best predict stimulus intensity. The proposal that a neuron's non-synaptic ion channels are selected by the temporal pattern of a neuron's input is reminiscent of the antigen-driven selection of antibodies found in the immune system. An analogous process would also occur in the spatial domain. In this case, the individual components could be GABA synapses, each synapse contributing prior information from a distinct point in space (determined by the presynaptic neuron's stimulus, or receptive field center). Those synapses from the surround that best predict stimulus intensity in the center would be strengthened [13].

To illustrate how this could occur, we again consider a neuron in which the second layer consists only of K+ channels, but now there are distinct types of K+ channels that vary in their kinetic properties. The conductance of the second layer (*G_K_*) at a given moment in time could be described as the weighted sum of the activities of each component (*i*) or type of K+ channel.
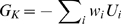
(2)The activity of a component (*U_i_*) refers to the time- and voltage-dependent likelihood that a channel of that type is open at a given moment (the channel's open probability in a Hodgkin-Huxley type model of average channel behavior). A component's weight (*w_i_*) would correspond to the number of functional channels of that type (in the formalism used here, weights would be negative for inputs contributing prior information (equation 3) and positive for inputs contributing current information (equation 4)). The weights could be adjusted by inserting or removing channels from the membrane, or by an event such as phosphorylation that could cause a channel to switch from one functional type to another [23], [24]. We would like to know the rules that govern the weights.

Minimizing the error means driving the membrane potential towards the middle of its range. If a depolarization-activated K+ channel is open when the membrane is depolarized, it is correctly guessing that glutamate concentration is high even though the neuron's second layer as a whole guessed too low. Therefore the weight of that type of K+ channel should be increased. If the membrane is hyperpolarized when a K+ channel is open, then channels of that type should be removed since they guessed too high and contributed to the negative error. If a K+ channel is closed, it bears no responsibility for whatever the voltage may have been, and its corresponding weight should not be changed substantially. These principles suggest a learning rule like the following:
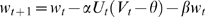
(3)where the weight of an individual component is updated at each moment in time (*t*) according to its activity (*U*), membrane voltage (*V*), and learning rates (*α* and *β*). The last term (*βw_t_*) would correspond to channels being removed from the membrane at a low rate, which would help to insure that the weight of channel types in which activity is not substantially correlated with membrane potential goes to zero. The term “*θ*” refers to a voltage near the middle of the range. It functions as the null point of membrane voltage where there is no error (Text S1). Depolarization beyond *θ* would increase weights, whereas hyperpolarization would decrease weights. For further details of the plasticity algorithm and mechanisms by which it might be implemented, see Text S1.

Plasticity algorithms such as equation 3 are often referred to as “anti-Hebbian.” A Hebbian rule strengthens depolarizing or hyperpolarizing inputs that are paired with depolarization or hyperpolarization, respectively, and it therefore involves positive feedback. An anti-Hebbian rule does just the opposite and results in negative feedback. Anti-Hebbian plasticity has been observed at both glutamate and GABA synapses, and it has previously been proposed to be involved in learning to make accurate predictions [12], 13,25–27. The present proposal extends its use to selecting amongst non-synaptic ion channels. A functionally relevant term for an anti-Hebbian rule would be “error minimizing.” Whereas some past work has emphasized the advantages of this type of plasticity, and adaptation in general, for efficient communication, the present work suggests how these phenomena help to solve the system's central problem, which is not to communicate inputs but to estimate their value.

### Hypothesis 4: Selection of Current Information Sources

*A neuron'ssources of current information (e.g. glutamate synapses) are selected to be those that are most closely associated with reward and the least predictable. One way this could occur is through a three-term Hebbian-type plasticity rule that incorporates reward feedback as well as pre- and post-synaptic activity.*

The principles discussed above could allow a system to predict the intensity of any sensory stimulus. However, real nervous systems are only concerned with those aspects of the world that are relevant to “future reward.” The definition of future reward used here is very similar to that found in the field of reinforcement learning, in which a key goal is to predict “the sum of future rewards” [2]–[4]. As described above under hypothesis 1, I propose that the general function of the nervous system is to predict (minimize uncertainty about) future reward. Future reward is ultimately defined in terms of biological fitness, or the future of an animal's genetic information, which is accepted to be the “goal” of all life forms. Thus the “future” necessarily spans generations. Since all of a nervous system's outputs should be selected to promote biological fitness, all the system's information should be about biological fitness (future reward). This broad and inclusive concept of future reward is quite abstract and intangible (as would be any attempt to specify the goal of life). However, as in reinforcement learning, the nervous system predicts future reward by predicting concrete physical stimuli that are themselves predictive of future reward. These stimuli would include every aspect of the world (internal and external) that can be sensed by the nervous system. For example, this would include generally weak predictors of future reward such as light intensity, as well as strong predictors such as the sight or taste of food. For a neuron in the “motor” system, the reward-predictive stimulus could correspond roughly to a “plan for action.” To use the language of animal learning theory, every stimulus can be thought of as a “conditioned stimulus,” although in some cases the “conditioning” has occurred over evolutionary timescales (in which case a stimulus would be genetically “hard-wired” and could be described as “unconditioned” with respect to the lifetime of an individual). Thus the concept of future reward, and the generality of the present theory, depend upon viewing the nervous system within the wider context of evolutionary biology.

Just as a neuron may select which points in space and time are most informative in predicting stimulus intensity, it may also select the stimulus that is most informative about reward. A prototypical neuron's proximal stimulus (its excitatory receptive field center) is defined as the glutamate concentration summed across a subset of synapses (in principle, a neuron's stimulus could instead be inhibitory). Those individual synapses in which activity is predictive of established reward predictors, such as food, should become strong. The selection process could be aided by an explicit reward signal. This could be provided by a neuromodulator such as dopamine [2], [3], [28] or it could come from the feedback projections that mediate selective attention in the neocortex. A reward feedback signal could be much less sophisticated than these examples, and in the simplest case it would be provided solely by natural selection over generations. Thus, at least in a wider biological context, there would always be some reward information present.

As described above, a stimulus should be selected for its correlation with reward. However, a second criterion is that to best predict future reward, a neuron should select the stimulus that is the least predictable (given the neuron's prior information). This is similar to the principle in statistics that the greater the variance in one parameter (e.g. light intensity), the greater its potential to explain the variance in another parameter (e.g. availability of water). However, even if the intensity of a stimulus has a high variance, and it is correlated with reward, it is not useful to a neuron if it is highly predictable, since it would merely be telling the neuron what the neuron already knows. Thus, other things being equal, it is the most unpredictable stimulus that would be expected to provide the neuron with the most information about future reward. Similarly, the most unpredictable stimulus has the most “potential,” or “exploratory value.” This is because even if no correlation has been identified between a stimulus and reward, such a correlation may be identified in the future, or by downstream neurons. For example, the selection of stimuli by neurons in some parts of the early visual system could be neither “hard-wired” nor guided by a dynamic reward feedback signal. In such cases, the selection of the least predictable stimulus would provide downstream neurons with the best chances of identifying a stimulus that is both correlated with a dynamic reward feedback signal and provides “new” (non-redundant) information.

A stimulus that is both predictive of future reward and unpredictable could be identified through application of a learning rule similar to that given above (equation 3), but with a sign change and now also including any reward information (*R*) that might be available:
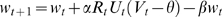
(4)If the only feedback about reward is provided by natural selection, then *R* would be constant over the lifetime of the organism, and this rule will simply tend to select the stimulus that is the least predictable. (Although not shown up above in equation 3, reward information may also shape the selection of inputs contributing prior information. However, even without a direct influence of reward in equation 3, the influence of reward in equation 4 will insure that a neuron's prior information is predictive of future reward.) In Text S1, I describe how the rule envisioned above could be implemented given our current understanding of synaptic plasticity mechanisms [29], and I further discuss the potential forms of the reward feedback signal. Although this rule tends to select channels and synapses in layer 1 that maximize the errors of layer 2 in predicting layer 1 output, it would tend to minimize the errors in the estimates of future reward by each of the two layers.

The error-maximizing plasticity algorithm of equation 4 is a “Hebbian” rule. As often pointed out, this type of rule strengthens synapses in which activity tends to be synchronous. Synchronous activation would occur more frequently in a subset of synapses that are driven by a recurring spatial pattern or “object” in the external world. Those synapses would become strong, thereby shaping the stimulus to which the neuron is tuned, as previously proposed [e.g. 14], [30]–[32]. The distinct proposal of the present work is that a Hebbian rule functions to maximize errors, and to suggest why this is advantageous in learning to predict future reward, the ultimate goal of the nervous system. An error-maximizing rule would help to insure that the stimulus contributes information about reward that the neuron does not already possess. For example, if a neuron receives a stereotyped temporal sequence of excitatory synaptic inputs, then the Hebbian rule will selectively strengthen the first input in the sequence (since prior information will tend to suppress responses to the latter excitatory inputs) (see ref. 14 for a similar proposal). Thus the error-maximizing rule explores the external environment to identify the best source of external information about reward, whereas the error-minimizing rule identifies the internal substrate that is best able to capture and hold that information. They both function together to maximize the neuron's information about future reward.

### Hypothesis 5: The System

*A network of the neurons described above, each neuron implementing the same types of plasticity rules, will organize itself into a system that performs the central function of accurately predicting future reward. In a mature system, each successive neuron leading away from the sensory periphery will have more information (less uncertainty) about future reward.*

The prediction of future reward is proposed to be the central function of the nervous system, and I have described above how this could be done by a single generic neuron. If each individual neuron performs this central function, it is relatively simple to describe how a system composed of these neurons could work together to better predict future reward. Because each neuron is at least roughly similar in its biophysical characteristics, we may presume that each neuron possesses a similar amount of information about its stimulus. However, some stimuli are highly informative about future reward (e.g. the sight of food), whereas others are only weakly linked to future reward (e.g. light intensity). Thus neurons differ in how much information they have about reward, and this is proposed to be the critical variable at the level of the system.

Because reward feedback contributes to the selection of each neuron's stimulus (equation 4), the stimulus of each successive neuron in a series progressing from the sensory to the motor peripheries would be more closely tied to future reward and less closely related to the immediate sensory world. Neurons further from the sensory periphery would therefore have more information and less uncertainty about future reward. This phenomenon can be illustrated by tracing a long path from the retina through the cortex to a motor neuron. In the visual system, the stimulus of successive neurons is transformed from small circles of light intensity to oriented bars and eventually to faces. Higher neurons in the parietal and prefrontal cortex are simply described as responding to “relevant information,” regardless of modality [33], [34]. Further along this path, neurons continue to become more selective for future reward, but they also become more “motor” by integrating proprioceptive and vestibular information specifically related to particular limbs and muscles. As the last neuron in the path, the stimulus predicted by a motor neuron would be very abstract and challenging to define precisely, but it could be described in rough psychological terms as a “plan for action.” Because the cumulative effect of reward feedback at every upstream synapse shapes the motor neuron's stimulus, the motor neuron would have less uncertainty about future reward than any of its upstream neurons. Likewise, the motor neuron would render the system's “decision.”

The amount of reward information possessed by a neuron and its stimulus also varies across sensory modalities. For example, because taste is more strongly correlated with future reward than is light intensity, a gustatory cell in the tongue has more information and less uncertainty about future reward than does a photoreceptor. Likewise, a gustatory cell is closer to motor neurons (separated by fewer synapses) than is a photoreceptor. If a gustatory and a visual path both converge upon the same motor neuron, then they would both produce the same reduction in uncertainty about future reward. However, because light intensity is a lesser predictor of future reward, the long path of the visual system must do more work than the short path of the gustatory system in order to cause the same reduction in reward uncertainty. However, in doing substantial work to extract reward information from light intensities, the long path of the visual system achieves a much greater reduction in uncertainty about the world in general.

### A Simulation

One approach to testing the current theory would be to simulate a network of these neurons. Previous studies have demonstrated how Hebbian [e.g. 30] or anti-Hebbian [13], [25], [27] synaptic plasticity rules could shape a network in accord with the present theory (although the proposed combination of both rules has not been simulated). The more novel aspect of the present theory, with regards to plasticity, is its application of the same sorts of plasticity rules to the selection of non-synaptic ion channels. I present here the results of a simulation in which a single compartment, graded potential, Hodgkin-Huxley type model neuron selected from amongst a spectrum of non-synaptic ion channels.

**Figure 3:**
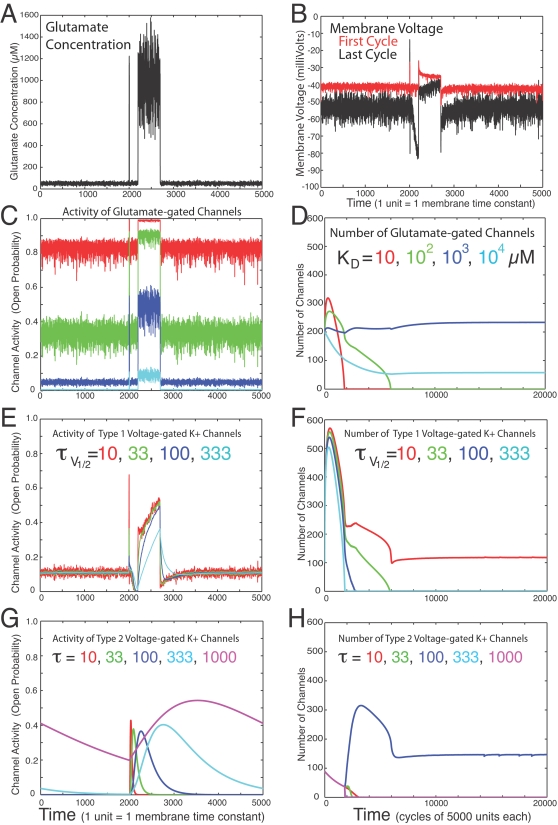
Selection of ion channels by plasticity rules. Hebbian (equation 4) and anti-Hebbian (equation 3) rules selected the channels of layers 1 and 2, respectively, in a single-compartment, graded-potential, Hodgkin-Huxley-type model neuron. See Text S1 for details. Initially, there were a total of 800 glutamate-gated non-selective cation channels in layer 1 (evenly divided among 4 subtypes) and 800 voltage-gated K+ channels in layer 2 (evenly divided among 9 subtypes). Other simulations began with different numbers and proportions of channels (not shown). The final numbers of channels were the same in all cases, regardless of the starting numbers (Table S1). A. At each time step, glutamate concentration was drawn from a Gaussian distribution with a standard deviation of 20% of the mean. The mean concentration increased from 50 to 1000 µM for 10 time steps starting at 2000, and again for 500 time steps starting at 2200. After 5000 time steps the pattern repeated, for a total of 20,000 cycles. B. Membrane voltage is shown for the first and last cycles. The average membrane voltage shifted towards the null point (θ = −50 mV) of the plasticity algorithms (equations 3 and 4). The hyperpolarization starting at 2000 in the last cycle was caused by activation of “type 2” K+ channels (see panel G) triggered by the first glutamate-driven depolarization. In the first cycles, these K+ channels were not activated because the first glutamate-driven depolarization was not large enough (see legend for panel D). The increased variance in membrane voltage in the last cycle was due to a decline in total membrane conductance together with an increased sensitivity of the glutamate-gated conductance to glutamate concentration. C. The activities (open probabilities) of the four types of glutamate-gated channel, each of which differed in its affinity for glutamate (KD). Each channel was gated by a single two-state glutamate sensor. D. The Hebbian rule (equation 4) selected primarily a glutamate receptor with moderate affinity (KD = 1000 µM, shown in blue). Elimination of high affinity receptors that were always near saturation increased the sensitivity of membrane voltage to glutamate concentration. The resulting increase in depolarization to the first pulse of glutamate allowed for activation of “type 2” K+ channels (see panel G). E. Activities of “Type 1” K+ channels during the last cycle. Each of four channel types, differing in their kinetic properties, was gated by a single two-state voltage sensor with a half-maximal activation at −40 mV. Maximal time constants (at −40 mV) ranged from 10 to 333 time units. F. Of the K+ channels in panel E, the anti-Hebbian rule (equation 3) ultimately selected the one with the fastest kinetics. Initially, the number of each type of K+ channel increased from its starting value of 89 because the membrane voltage was almost always depolarized beyond θ (−50 mV). G. The activities of “type 2” K+ channels during the last cycle. These channels each consisted of 2 layers of 4 sensors each. The sensors of the first layer were not modeled realistically, but instead were all “turned on” instantly whenever the membrane was depolarized beyond −25 mV. They then turned off slowly. The sensors of the second layer adapted to those of the first layer with kinetics that varied across channels as shown. Each channel was open only when at least one sensor in layer 1 was on and all sensors in layer 2 were on. H. The anti-Hebbian rule selected the type 2 K+ channel with intermediate kinetics (τ = 100). This channel was able to use the first pulse of glutamate to predict and partially cancel the effect of the second pulse of glutamate. Initially, the numbers of all type 2 K+ channels declined because membrane voltage never exceeded the threshold necessary to activate them.

The simulated neuron simultaneously selected from amongst four subtypes of glutamate-gated cation channels in layer 1 through a Hebbian rule (equation 4), and from amongst nine subtypes of voltage-regulated potassium channels in layer 2 through an anti-Hebbian rule (equation 3). The stimulus was glutamate concentration, which was drawn from a Gaussian distribution at each time point (Fig. 3A). The mean concentration increased during two square wave pulses (the first of which was very brief). This pattern repeated itself for a total of 20,000 cycles. The final number of channels of each of type appeared to have little or no dependence on the starting numbers (Table S1).

The four types of glutamate-gated channels of layer 1 differed in their affinities for glutamate (Fig. 3C). Subtypes with intermediate affinities were the most sensitive to the actual range of glutamate concentrations to which the neuron was exposed. Their activity was more variable and less predictable, and one of these was therefore the predominant subtype selected by the Hebbian plasticity rule (Fig. 3D). As learning progressed and higher and lower affinity receptors were eliminated (Fig. 3B), the neuron's membrane potential became more sensitive to glutamate concentration (Fig. 3B, compare last cycle in black to first cycle in red).

Layer 2 consisted of four “type 1” and five “type 2” K+ channels. Type 1 channels were gated by a single sensor, and thus their predictions of membrane voltage (and glutamate concentration) were simply an exponential function of past voltages. There were four subtypes of type 1 channels, each with a different time constant (Fig. 3E). The anti-Hebbian rule of equation 3 selected the type 1 channel that had the fastest kinetics, since this channel was best suited to exploiting the correlations in voltage created by the membrane time constant, and it was also the channel that was able to adapt its prediction most quickly to the step changes in mean glutamate concentration. In the absence of these patterns, the channel with the slowest kinetics was favored, since that channel made the best predictions by averaging over the longest period of past voltages (not shown).

Each of the five subtypes of type 2 K+ channels was gated by eight sensors and differed from the other subtypes in its kinetics. The gating of each type 2 channel by multiple sensors made it more like real channels (relative to the “one-sensor” type 1 channel described above). However, the rules of channel gating (see figure 3 legend and Text S1) were chosen so that a channel opens for a certain time period (specified by the kinetics of its sensors) after a sufficiently large depolarizing event (Fig. 3G). Thus a type 2 channel could use the first pulse of glutamate to predict and counteract the depolarization caused by a second pulse of glutamate (Fig. 3G). Indeed, the subtype of type 2 channel selected by the anti-Hebbian rule had kinetics that roughly matched the actual interval between the two pulses of glutamate (Fig. 3H). This subtype was selected because it counteracted the glutamate-driven depolarization (positive error), and in spite of the fact that it also caused a brief hyperpolarization (or negative error) prior to the second pulse of glutamate (Fig. 3B, data in black). Text S1 provides details of the simulation, as well as additional discussion of the temporal predictions made by ion channels

## Discussion

Perhaps the most compelling aspect of the present theory is its simplicity. Each neuron is proposed to perform the same basic computational function, selecting its inputs according to the same principles in order to better predict an aspect of the world related to future reward. However, a neuron's information and connectivity will develop differently from other neurons due to the particular statistical pattern of inputs to which it has been exposed. The theory is grounded in well established and universal biophysical properties of neurons, and it is at least consistent with what is currently known about neuronal plasticity. Beyond providing a plausible explanation of how the nervous system could perform its central function, a critical measure of the theory's value will be its ability to predict the synaptic connectivity and intrinsic membrane properties of neurons given knowledge of the statistical structure of their inputs. Much of the strongest evidence in this regard naturally comes from those parts of the nervous system that are the best understood, the early sensory systems.

The theory suggests that some of a neuron's inputs should be selected in order to maximize the neuron's prediction errors (deviations in membrane potential), whereas other inputs should be selected to minimize errors. In some types of neurons, the selection could have occurred over evolutionary timescales through natural selection. But other neurons are presumed to exhibit plasticity that would allow them to select their inputs according to the particular statistical patterns to which they have been exposed. In a typical neuron, excitatory synaptic inputs provide the neuron with current information about the stimulus to which the neuron is tuned, and these inputs are proposed to be selected according to a Hebbian or error-maximizing rule. This proposal represents the mainstream view of how stimulus specificity develops [30]–[32]. These inputs typically correspond to glutamate synapses, where Hebbian plasticity is a well-established phenomenon [29]. The present proposal builds on previous work by suggesting why Hebbian plasticity helps neurons to perform the system's ultimate function of predicting future reward.

Most other inputs to a plastic neuron are proposed to be selected through an anti-Hebbian rule. These inputs contribute prior information, and would include (among others) inhibitory synaptic inputs and voltage-gated potassium channels. Little is known about the rules and mechanisms by which a neuron selects amongst these inputs. However, although rather indirect, there is substantial evidence for an anti-Hebbian type rule in the extensive literature on “efficient coding,” which dates back approximately 50 years to the work of Attneave [35] and Barlow [9]. The basic principle is that neurons should not signal predictable components of the world, because it would be redundant and wasteful to tell the system what it already knows. Obviously this requires that the system has prior knowledge about the world, and it has previously been proposed that an anti-Hebbian plasticity rule would function to select the best sources of prior information [25], [27]. The best sources of prior information would be those that most effectively counteract excitation and inhibition of a neuron so that its membrane voltage signals only the unpredicted component of the neuron's stimulus. The widespread phenomena of adaptation and surround inhibition support this model. For example, there is a positive correlation between the light intensity of different colors, and a particular type of retinal ganglion neuron is excited by blue light and inhibited by red-green light, effectively signaling errors in the prediction of blue light [21]. One would expect that, in many cases (but not all), the retinotopic or tonotopic region that is the best predictor of a stimulus would be similar in spatial extent to the region that constitutes the stimulus itself, and likewise the tuning of a neuron's synaptic inhibition has been found to be similar to the tuning of its excitatory stimulus [e.g. 17]–[20]. Furthermore, there is evidence that the rate of adaptation and the spatial extent of the surround can be dynamically selected to better predict and cancel the excitatory effect of the stimulus [e.g. 13], [36]. Direct evidence that anti-Hebbian synaptic plasticity does in fact mediate the selection process has come from work in retina [13] and in cerebellum-like structures [12], [26].

A particularly novel aspect of the present theory is the proposal that many of a neuron's non-synaptic, intrinsically gated ion channels may be selected in order to minimize prediction errors, or deviations in membrane voltage. One way this selection could occur is through anti-Hebbian plasticity. There is a large diversity of voltage-regulated potassium channels that differ in their kinetic properties. By selecting amongst these channels, an anti-Hebbian rule would be selecting those periods of the past that are the best predictors of current stimulus intensity. If the theory is generally applicable, then the principle of minimizing prediction errors should be able to explain, for example, the finding that hair cells tuned to higher frequencies of mechanical stimulation express voltage-dependent potassium channels with faster kinetics [37] (although this could be genetically specified in the case of hair cells, rather than achieved through anti-Hebbian plasticity). Previous studies have shown that the selection of channel types can be activity dependent, and it has been proposed that this plasticity has a homeostatic function in stabilizing membrane potential [e.g. 38]–[40]. The present proposal is consistent with such past work, but suggests a more sophisticated computational role for these channels. Implementation of the proposed anti-Hebbian rule would require detecting not only neuronal activity, but the coincidence between neuronal activity and channel conformation or conductance. Thus, as described in Text S1, the regulation of these channels would require a mechanism that is similar in its complexity and sophistication to that found postsynaptically at glutamate synapses [29]. There is currently very little direct evidence for or against this proposal, which could be tested by examining the influence of different patterns of neuronal activity on the kinetics of a neuron's potassium channels.

The primary obstacle to confirming or rejecting the present theory, and particularly hypotheses 3 and 4, is our ignorance of the statistical structure of the world. Progress has been made in quantifying the statistical patterns that are relevant to early sensory systems, and in relating those patterns to the properties of early sensory neurons [41]. Indeed, much of the evidence for the present theory comes from early sensory systems. However, quantifying relevant statistical patterns is a difficult undertaking even for early sensory systems, and studies of natural stimulus statistics have not yet provided much insight into the function of neurons beyond primary sensory cortices. According to the present theory, later neurons function according to the same principles as earlier neurons. The success of the theory in accounting for the function of early sensory systems supports its application to neurons at later stages of processing, since there do not appear to be substantial and consistent differences at the cellular or molecular level between “higher” and “lower” neurons. However, it is very difficult to characterize the statistical pattern of inputs to a neuron far from the sensory periphery, since its external stimulus is difficult to define precisely, and its proximal stimulus (excitation summed across a subset of synapses) can't be measured with present technology. As an alternative to quantifying a neuron's natural stimulus statistics, *in vitro* experiments can provide control over the pattern of a neuron's inputs. Such work has focused primarily on spatial patterns (i.e. correlations between synapses), and has provided evidence for Hebbian and anti-Hebbian synaptic plasticity [e.g. 12], [13], [26], [29] [Bibr pone.0003298-Bienenstock1] [Bibr pone.0003298-Bear1]–[32]. However, very little has yet been done to manipulate the temporal pattern of a neuron's input in order to test whether its non-synaptic ion channels are selected according to an anti-Hebbian rule (hypothesis 3). Thus our lack of knowledge of the statistical structure of a neuron's inputs limits our ability to judge the proposed relationship between the pattern of those inputs and their selection by neuronal plasticity.

The present theory can be seen as a synthesis of the fundamental principles of reinforcement learning with those of efficient coding. The “efficient coding” hypothesis has been among the most successful of all computational approaches in explaining the function of the nervous system [6], [41]. However, the principle of efficient communication does not address the general function of the nervous system. Through its reliance on prediction errors, reinforcement learning also incorporates the principle of efficiency, and unlike efficient coding, it does address the general function of the system (although not in the mathematically precise sense described above in hypothesis 1). However, in comparison to efficient coding, reinforcement learning has only more recently been applied to understanding the nervous system, and this effort has largely been restricted to relatively high level sensorimotor systems and behavior rather than generic single neurons and early sensory systems. The present theory incorporates the function of early sensory systems and principles of efficient coding within the more general framework provided by reinforcement learning. However, the distinct approach to information and probability taken above in ‘hypothesis 1’ is critical to the claim that the present theory addresses the computational goal of the nervous system. By contrast, the literature on reinforcement learning has generally not incorporated quantitative notions of information, and most of the literature on efficient coding has applied a definition of probability that cannot be used to quantify the information found within the nervous system (Text S1).

A key question regards the generality of the proposed model. Does the computational goal, outlined under hypothesis 1, really apply to all neurons and all systems? I have proposed that minimizing uncertainty about future reward is the goal of all neural systems, and I have suggested how this could be accomplished. ‘Future reward’ is defined here (see hypothesis 4) in such a broad and abstract sense that it should apply to all neural systems. It is better thought of in terms of biological fitness than in terms of concrete reward stimuli such as liquid volume. Likewise, the relevance of the system's information to future reward is ultimately insured through natural selection. Hebbian and anti-Hebbian rules are moderately sophisticated methods of selecting amongst inputs (information sources) to achieve the goals of hypotheses 3 and 4, but natural selection over generations could achieve a similar computational goal. Thus the theory does not require that all neurons display plasticity (in the usual sense of the term).

Another potential challenge to the generality of the theory comes from the great diversity of neuronal types. Is this diversity compatible with the proposal that all neurons share a basic computational goal? The theory seeks to explain some aspects of neuronal variation that are clearly relevant to computational function. It suggests how synaptic connectivity would reflect the spatial structure of the external world, and how a neuron's dynamic membrane properties would reflect the temporal structure of the world. Thus the theory could explain how different neurons come to have different information. However, one can easily imagine that the theory may hold true for some types of neurons but not others. For example, although inhibitory neurons are fundamentally similar in most respects to excitatory neurons, there may be important differences that are not addressed by the present theory. Furthermore, the present theory has largely ignored the need for reliable communication across distances (instead treating neurons as single compartments), a need which may explain the prevalence of axonal and dendritic action potentials (see Text S1), and which may be a major factor in determining cell morphology. Another important factor that has not been addressed concerns metabolic constraints, which could, for example, limit channel density and connectivity with distant neurons. Finally, some aspects of neuronal variation would presumably be irrelevant to computation, such as the specific molecular identity of a neurotransmitter. Thus, even if the theory proves to be general in the sense described above, it would only be expected to account for a portion of neuronal variation.

The main focus here has been on presenting a theory of biological nervous systems, and thus a key question is how well the theory is able to predict the structure and function of the nervous system given the statistical structure of its inputs. However, the theory also suggests a computational framework that could be useful in designing artificial neural networks. An important test of both the biological plausibility and computational utility of the present theory would be to examine the characteristics of an artificial network of the proposed model neurons. If correct, a network of generic model neurons should be able to organize itself so as to generate “intelligent” or “rewarding” outputs. Ideally, each neuron would initially differ only in its spatial location within the network and in the sign of its output (excitatory or inhibitory, or “modulatory” in the case of a reward signal such as dopamine). The hypothesis is that such a system will be able to organize itself appropriately (depending on the structure of its inputs) without substantial additional information being “built-in” from the start. (This is not to suggest that the development of real nervous systems is entirely dependent on the environment to which the individual is exposed, since development is clearly instructed by genetic information as well. But if the theory is correct, then the plasticity rules implemented in an artificial network may converge on solutions or architectures that are similar to those reached through biological evolution (as well as through development and learning within an individual).)

Although the neurons described here are certainly no more complex than real neurons, they are quite knowledgeable and sophisticated in comparison to the neurons of typical neural network models. In such models, most if not all of a neuron's information is carried in its synaptic weights and electrical output, whereas non-synaptic ion channels often play no role [42]. By proposing important roles for many of the diverse types of ion channel that are known to exist in real neurons, the model neuron described here could be thought of as a network unto itself. Most of the neuron's information is in chemical form, and it is not stored only in synaptic weights. Membrane voltage represents only the errors in prediction, which serves to update the chemical information of local and downstream neural elements. If the theory is correct, then this highly efficient means of communication, together with the vast information capacity associated with a large number and diversity of molecules, should make the single neuron modeled here more “intelligent” than the typical neurons of network models.

Beyond describing a model neuron that may have significant computational advantages, the present theory has approached the problem from a fundamentally different perspective. Most work on neural networks has focused on creating networks that generate desired outputs, with relatively little concern for what information the network contains or how its information is organized. By contrast, the present theory focuses on the information contained by neurons and networks, and proposes that desirable outputs will follow in a simple and natural manner if the system has the appropriate information. (To understand how this is possible, recall that the system's output shapes its information through reward feedback.) The theory suggests that a system's information determines its output, and that the more information the system has about future reward, the more advantageous its output will be. The proposal that to predict (and thus “understand”) the world is virtually sufficient for selecting appropriate outputs is based on the simple argument that the best output is always obvious in the absence of uncertainty. Simulations of networks of these neurons will be necessary to test whether the abstract goal of minimizing uncertainty about future reward really is sufficient for maximizing reward in the concrete sense that has been the standard of most past work.

## Methods

### Calculation of Conditional Probability Distributions

Ion channels typically consist of several protein subunits, and each subunit alternates between multiple configurations, or states [22]. A protein divides its time between states according to their relative energies, spending more time in states of lower energy (Fig. S1). The likelihood that a protein is in a state with a given energy is specified by the Maxwell-Boltzmann equation of statistical mechanics [22]. If a protein has just two possible states, the probability *P_2_* that the protein is in state 2 depends on the energy difference (*E_2_*–*E_1_*) between the two states,
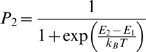
(5)where *k_B_* is Boltzmann's constant, and *T* is temperature in Kelvin. If the energy difference is dependent on a quantity such as voltage or the binding of a ligand, then the protein molecule possesses information about that quantity and it functions as a sensor. For simplicity, the ion channels in the simulation of figure 2 were gated by single two-state sensors.

For a voltage sensor, the energy difference between the two states in the Maxwell-Boltzmann equation (equation 5) is
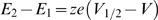
(6)where *z* is the number of equivalent elementary charges, *e* is the elementary charge in coulombs, *V* is voltage, and V_1/2_ is the voltage required to counterbalance the inherent energy difference between the two states so that they are equally probable. The sensor for a chemical works in a similar but slightly different manner. Binding of a ligand acts to stabilize state 2 of the sensor. However, unlike the dependence of a sensor's energy states on voltage, the relative energies of the bound and unbound states are independent of ligand concentration. The exponential term in equation 5 is thus a constant (for a given temperature), and the likelihood (*P_2_*) of a receptor being bound turns out to be
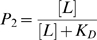
(7)where [L] is ligand concentration and K_D_ is the equilibrium dissociation constant. Equations *5*–*7* specify the likelihood that a single ligand or voltage sensor is in the “*on*” or “*off*” conformation as a function of stimulus intensity, as shown in figure 2A.

Bayes's theorem describes how information should be integrated across multiple sensors arranged in parallel or in series, as described in detail in Text S1. It was assumed that the sensors function independently of one another, and thus the likelihood functions associated with single sensors (Fig. 2A) were simply multiplied together to derive the probability distribution conditional on the whole population of sensors (Fig. 2E–H).

### Simulations

See Text S1 for details. The simulations of figures 2 and 3 were performed with Matlab and were based on a single-compartment, graded potential, Hodgkin-Huxley type model neuron. Whereas the simulation of figure 2 had a fixed number of ion channels, the simulation of figure 3 implemented the plasticity algorithms of equations 3–4 to modulate the number of ion channels of various types. The value of *θ* (equations 3 and 4) was chosen to be −50 mV.

## Supporting Information

Text S1 Supporting Information Text
(0.10 MB DOC)

Figure S1 Illustration of a sensor
(0.33 MB EPS)

Table S1 Final numbers of channels of each subtype in the simulation shown in Figure 3.
(0.35 MB EPS)
