# Supplementary material for: Towards a General Theory of Neural Computation Based on Prediction by Single Neurons
Source: PLoS One. 2008 Oct 1;3(10):e3298. doi: 10.1371/journal.pone.0003298 (PMC2553191; doi:10.1371/journal.pone.0003298)
Supplement: Text S1 — Supporting Information Text (0.10 MB DOC) [file pone.0003298.s001.doc]

#### **SUPPORTING INFORMATION – TEXT S1**

# INTRODUCTION

# The Definition of Probability and Its Application to the Nervous System

Hermann von Helmholz argued in the 19^th^ century that the nervous system must infer or estimate the structure of its environment, and this ‘constructivist’ view of neural function is commonly held today. By definition, an estimate involves uncertainty and is therefore properly described in terms of probabilities. Although probability theory is widely used to describe neural function, contradictory definitions of probability have been proposed, and there are different approaches that can be taken in applying the concept of probability to the nervous system. The present work suggests that a particular approach to probability theory may provide a simple and compact description of neural function.

Although seldom acknowledged, two fundamentally distinct definitions of probability have been proposed. These can be illustrated by the differing answers that might be given to a simple question: “An event has four possible outcomes, *A, B, C*, and *D*. What is the probability of each outcome?” The “Bayesian” answer is that, in the absence of any additional information, logic requires that all outcomes be considered equally likely, and thus the probability of each is 0.25. The “frequentist” response is that the question is inappropriate. In order to apply the concept of probability, one must first observe the frequency with which each outcome occurs. As one makes repeated observations of the outcome, one can begin to “estimate” the probabilities. To be certain of the probabilities, one would have to make an infinite number of observations. According to a strict frequentist definition, probabilities are equivalent to frequencies; they are a physical property of a system, and they are independent of an observer’s knowledge of the system. This is the view taken by Feller (1950) in his classic work on probability theory. Most work in neuroscience implicitly accepts elements of the frequentist definition, without explicitly addressing the definition (e.g. Rieke et al., 1997; Dayan and Abbott, 2001).

The Bayesian definition of probability has been advanced by many authors over the years, but the account given here is based upon the recent text of Jaynes (2003). According to this view, probability theory is a natural extension of earlier work on logic. By insisting that propositions be either true or false, conventional logic is not applicable to the conditions of uncertainty that characterize nature. Probability theory incorporates uncertainty by describing confidence (or strength of belief) in a proposition on a continuous scale of 0 to 1. The probability of a particular proposition (or event) is always entirely conditional on a particular set of knowledge or information. The rules relating knowledge to probability are essentially just “common sense” (at least for simple states of knowledge). Indeed, Laplace (1819) referred to probability theory as “nothing but common sense reduced to calculation.” Much of what we mean by common sense is embodied mathematically in the principle of maximum entropy. Entropy is a measure of the uncertainty inherent in a probability distribution, and it increases with the width or flatness of the distribution (Shannon, 1948). The maximum entropy principle (or logic) requires that we fully acknowledge our ignorance by considering all possibilities equally probable unless we have evidence to the contrary. For example, if the only information available is that “there are four possible outcomes,” then the probability distribution that describes that information is “flat” since entropy is maximized when the probabilities are all equal. Since by definition the sum of the probabilities must equal one, the probability of each outcome is 0.25. In contrast to this contrived example, we often have information that does not constrain the number of possible outcomes, but does indicate that some outcomes are more probable than others. Such a state of knowledge often corresponds to a Gaussian probability distribution, which has the maximum entropy for a given variance. In other cases our knowledge derives from observing the past frequency of an event, in which case the probability distribution that best describes our knowledge may closely resemble the observed frequency distribution. If two rational entities possess different information, they must assign different probabilities to the same event. In this sense, probability and uncertainty are subjective and relative rather than objective or absolute properties of the world. However, a given state of knowledge uniquely determines a single probability for a particular event through the principle of maximum entropy (or logic). Therefore, any two rational entities possessing exactly the same knowledge must assign the same probabilities to all events, and in this sense probabilities are objective properties of an information state.

Scientists typically use probability theory without stating a formal definition of probability, and in practice they often incorporate elements of both the Bayesian and frequentist definitions. This is illustrated by the commonly used notation of Bayes’s theorem, an equation which specifies how distinct sets of information about an event ought to be rationally integrated to yield an optimal estimate. Whereas the “posterior” probability is explicitly stated to be conditional on a set of information, the conventional notation implies that the “prior” probability is not conditional on any information but is instead equivalent to a frequency distribution (e.g. Rieke et al., 1997; Dayan and Abbott, 2001). The strictly Bayesian approach taken here insists that all probabilities are entirely conditional on a set of information. When it has been implied elsewhere that probabilities are unconditional, the authors are simply not acknowledging explicitly what information they used to determine the probability distribution.

Does the definition of probability really matter? Don’t the ‘numbers’ work out to be virtually identical regardless of the definition? Indeed, probability theory has proven itself useful to scientists without there being agreement on its definition. The precise definition of probability is *usually* not of practical importance because scientists usually seek to describe the world based on a common body of knowledge. If two scientists base their estimates on shared information, the probabilities will be identical. In contrast, two nervous systems routinely possess very different information about the same event, and therefore the probabilities may differ greatly between the two perspectives. Thus a Bayesian understanding of probability provides us with two equally valid but very distinct approaches to describing neural function. We can either describe our information about a nervous system and its environment, or we can describe a nervous system’s information about its environment.

The conventional approach is to use probabilities to describe our information as scientists about events such as a neuron’s spike output, or vesicle release. In their rigorous investigation into the information carried by a neuron’s spike output, Rieke and colleagues (1997) also use probabilities to describe their own information, but in a slightly different manner which they refer to as “taking the neuron’s point of view.” In a typical experiment, they present a neuron with a sensory input of continuously varying intensity, and their goal is to estimate the current stimulus intensity at each moment by observing the neuron’s spike output while ignoring their direct observation of the current intensity. Their knowledge of the frequency distribution of stimulus intensities provides them with their prior probability distribution (although they don’t explicitly acknowledge that the prior is conditional on any information). That prior information is then integrated with their observation of the neuron’s spike output in order to estimate current stimulus intensity. Although their observation of the neuron’s spike output informs their estimates, those estimates are nonetheless entirely based on their knowledge as scientists. They do not address what information is actually possessed within the biophysical substrate of the neuron. The current work attempts to take the neuron’s point of view by asking “what is the probability distribution of potential input values conditional *only* on information integrated within the neuron’s membrane potential?” To the best of my knowledge, this question has not been asked before.

Although the present approach of taking the neuron’s point of view represents a departure from past work in neuroscience, it is not much different from how we as individuals, outside of science, try everyday to understand and predict the behavior of other people. Tremendous progress in predicting the actions of others is made in the development of a child, and in the evolution of intelligent species, with the realization that the actions of another individual are usually guided by distinct knowledge about the world that differs from one’s own knowledge (“a theory of mind”). To predict the actions of other people, one tries to “see the world through their eyes” by inferring their state of knowledge. To the extent that one knows what information another person possesses, that person’s behavior can often be accurately predicted. Of course when we do this everyday we do not use probability theory in a formal sense. However, the Bayesian understanding of probability lays out the principles by which we could attempt to give a formal mathematical description of another person’s knowledge. Indeed, recent studies have helped us to understand phenomena of human psychology and behavior, such as perceptual illusions and language, by using probability theory to describe an individual’s knowledge from their first-person perspective (Knill and Richards, 1996; Seidenberg, 1997; Weiss et al., 2002; Rao et al., 2002; Yang and Purves, 2003; Singh and Scott, 2003; Purves and Lotto, 2003; Niemeier et al., 2003; Kording and Wolpert, 2004). The rationale for trying to adopt the neuron’s first-person point of view, rather than the conventional third-person perspective of the scientist, is that the first-person perspective may provide us with a simpler, more general description of neural function, in much the same way that a theory of mind facilitates our understanding of other people.

# METHODS

#### **Probability Distributions Conditional on the Information in a Single Sensor**

Here I describe in greater detail how the information contained in a single sensor specifies a probability distribution of potential stimulus intensities. To infer the precise probability distribution requires that we have complete knowledge of the biophysical rules that govern the sensor, but we should be able to make reasonable estimates in the case of simple systems. Given equations *5-7*, one can determine the probability distribution of voltages or ligand concentrations given the state of one or more sensors. The knowledge of a sensor can be split into two components. First, it knows how the likelihoods of the two states vary depending on the stimulus. This is an inherent property of the sensor that does not vary over time, as described by equations *5-7* and illustrated by the energy diagram of supplemental figure 1. Second, the sensor knows its current state (but not past states). Although the probability distribution may be readily apparent given this simple set of information, we will nonetheless derive it in a general manner using Bayes’s theorem, which describes how information is to be integrated (Jaynes, 2003).

(8)

In this example, information *B* and *C* are being integrated to specify the probability of *A (pA* | *BC*) (the “posterior” distribution)*.* Let *A* be the stimulus intensity (voltage or ligand concentration), *B* the “*on*” state of the sensor (favored by high intensities), and *C* all the knowledge of the sensor except for its current state, including knowledge of how the likelihood of *B* varies with *A*. The “likelihood function” *(pB* | *AC*) specifies the probability of *B* for each possible value of *A*. As stimulus intensity increases, the *on* state of the sensor becomes more likely. Thus if one observes the *on* state, one can guess that higher intensities are more likely. Of course the estimate also depends on any prior information that is available (*pA* | *C*). However, in the case of a single sensor, there is no prior information about the current intensity, and thus all possible intensities would be equally probable “prior” to observing the state of the sensor. The probability of *B | C* would be equivalent to the sum of the product of *pA | C* and *pB | AC*, which is simply the normalization factor needed to insure that *pA | BC* is a probability distribution. At the microscopic level discussed here, the “intensities” could be considered to have discrete values. The range of possible intensities could be considered infinite, but as a practical matter we can arbitrarily choose it to be sufficiently large so as to exceed natural variation. Having specified a range and a “unit” size, the prior distribution then becomes one divided by the number of possible intensities. For the case of a single sensor, Bayes’s theorem simply indicates that the probability distribution has the same shape as the likelihood function (Fig. 2A).

Together with the current state of the sensor, knowledge of a sensor’s activation energy (Supplemental Fig. 1) entails information about past and future stimulus intensities as well. The activation energy (*E_a_*) determines how long the current state of the sensor is likely to last, as described by the Arrhenius equation (which is derived from the Maxwell-Boltzmann equation (equation 5)),

(9)

where *k* is the rate constant and *A* is a constant sometimes called the attempt frequency. The likelihood of a two-state sensor being in its current state would decay exponentially from 1.0 to 0.5 with a time constant of *1/k*. The probability distribution of stimulus intensities at a time in the past or future would be the average of the two alternative probability distributions (*P_1_* and *1-P_1_*), each weighted by its time-dependent likelihood. Thus the relatively steep probability distribution at time *t* = 0 (Fig. 2A) would gradually become flat as future or past time is projected to infinity.

**Probability Distributions Conditional on the Information in Multiple Sensors**

A cell consists of a very large number of sensors, and even a single protein molecule usually contains multiple sensors. Sensors can be arranged either in parallel or in series. A parallel set of sensors, such as all of a photoreceptor’s functional rhodopsin molecules, will be referred to as a “layer.” The estimate of stimulus intensity by a population of sensors within a single layer can be calculated by considering each sensor in turn through multiple iterations of Bayes’s theorem. Each additional sensor would further reduce the uncertainty in the layer’s estimate (Fig. 2A,E-H). In the absence of any knowledge of an interdependence or correlation between two sets of evidence (“*B*” and “*C*” in equation *8*), Bayes’s theorem states that probabilities should simply be multiplied. Empirical evidence indicates that discrete ion channels usually function independently of one another, and even subunits within a single ion channel may function in a relatively independent fashion (Hille, 2001). However, if they were not independent, then the ion channels (or molecules with which they interact) would necessarily possess information about the dependent relationship, and Bayes’s theorem would instruct us in how to proceed.

A second layer of sensors would act to “sense” and predict the state of the first layer. The second layer could be within the same protein molecule. For example, a subunit of a glutamate-gated ion channel could consist of two layers with one sensor each. The first layer would have a glutamate sensor, or binding site, which permits current to flow when it is in its bound state. When the sensor of the second layer guesses that the first sensor is in its bound state, it would stop current from flowing, and it would therefore mediate desensitization. Although the second layer has no direct interaction with glutamate, it can nonetheless estimate glutamate concentration by estimating the state, or output, of the first layer. If we let the estimate by the first sensor be either the probability distribution *P* or *1- P*, depending on whether it is in state 1 or 2, then the estimate by the second sensor would be the weighted average of *P* and *1-P*, where the weights would be the likelihood that the first sensor is in the corresponding state (conditional on the information of the second sensor). It should be noted that because a deeper layer has no direct interaction with the stimulus, its estimate of stimulus intensity must depend on knowledge of how the stimulus interacts with more superficial layers. Thus, for us to specify the probability distribution of potential input intensities to layer 1 conditional only on the information of a deeper layer *X*, requires that we have knowledge of the system (layers 1 through *X*) that is not local to layer X itself.

We can apply the same reasoning to determine the information contained within a more realistic ion channel. For example, a voltage-activated channel could be made up of 4 identical, independently functioning parallel subunits, each of which contains a voltage-sensitive activation gate in layer 1 and a voltage-insensitive inactivation gate in layer 2. It would only be open and conducting when all sensors of the first layer are *on* and all sensors of the second layer are *off*. As described above, information can be integrated across the parallel sensors within a given layer using Bayes’s theorem. However, we would like to know the distribution of potential voltages conditional only on the conductance of the channel and knowledge of the rules that govern it, but without certain knowledge of the current configuration of its multiple sensors. If *P* and *1-P* denote the probability distributions associated with the *on* and *off* states, respectively, of a single layer 1 voltage sensor, then the open channel would indicate that the distribution of potential voltages is *P*^4^. This is because all 4 sensors of the first layer must be *on*. The sensors of the second layer must all be *off*, but this knowledge is irrelevant since the second layer merely represents an estimate of the first layer’s conformation, which is already known with certainty. In contrast to the open channel, the closed channel could result from multiple combinations of sensor states. The estimate of voltage conditional on the closed channel would therefore depend on the probability-weighted sum of the distributions associated with each possible conformation. Thus computation of the probability distribution conditional on the closed state would be more complex than the case of the open state. Whereas an open channel would correspond to a steep sigmoidal probability distribution strongly favoring positive voltages, a closed channel would correspond to a relatively flat distribution slightly favoring negative voltages.

## **Details of the Simulation of Figure 2**

Figure 2 illustrates the glutamate probability distributions conditional on the state of the sensors in layers 1 and 2 of a single-compartment, graded-potential Hodgkin-Huxley type model neuron. The simulation was run on Matlab. There were 100 glutamate-gated channels in layer 1 and an equal number of voltage-gated channels in layer 2, as described in the legend of figure 2. Each channel was gated by a single two-state sensor. Unlike the simulation of figure 3, the number of channels was not altered by a plasticity rule.

The membrane voltage approached its steady state value ( in equation 1) according to:

(10)

## where the membrane time constant (τ_M_) was fixed at one. Although the simplification of a fixed membrane time constant was not of importance here, it was useful in the simulation of figure 3 (see below).

The conductance of each layer was allowed to vary continuously. The steady state conductance of glutamate-gated nonselective cation channels (layer 1) was determined by equation *7* with K_D_ = 500 µM. The conductance of glutamate-gated nonselective cation channels followed reversible second order kinetics (Boecker, 1984). Although the use of second-order kinetics made this simulation more realistic, it was not necessary or relevant to the purpose of the simulation.

The steady state conductance of K+ channels () was determined by equations *5* and *6*, with *V*_1/2_ = -50 mV and *z* = 4. Whereas real K+ channels, like those of the original Hodgkin-Huxley model, might be gated by four subunits, these were gated by just a single subunit, making the channel behave as a simple two-state sensor. This was done for the purpose of presenting the simplest relationship between sensor state, channel conductance, and the corresponding probability distribution. The K+ conductance approached its steady state value with reversible first-order kinetics:

(11)

The time constant (τ) was computed as described by Keynes and Rojas (1974) for a single two-state voltage sensor:

(12)

where $t_{\text{Max}}$ was the time constant when *V = V_1/2_*, which is the voltage at which the time constant has its maximal value. It was arbitrarily chosen to be 400 time units. “*k*” in equation *12* is a constant:

(13)

z = 4 (gating charge, chosen to be in the range of the gating charge of an actual voltage-sensitive channel)

e = 1.602 x 10^-19^ (elementary charge in Coulombs)

k_B_ = 1.381 x 10^-23^ (Boltzmann's constant in Joules /molecule / Kelvin)

T = 298 (Temperature in Kelvin; 298 K = 25 Celsius)

Although membrane voltage and conductance were allowed to vary continuously, discrete possible values of glutamate, glutamate-gated conductance, and membrane voltage were used to calculate probability distributions. Possible glutamate concentrations were integer values from 1 to 10,000 µM, glutamate-gated concuctances were 0.1 to 100 units in increments of 0.05, and voltages were –100 to 0 mV in 0.5 mV increments.

## **Details of the Simulation of Figure 3**

The simulation that produced figure 3 was also run on Matlab. It demonstrates how a neuron could select its ion channels in order to more efficiently and accurately predict its stimulus. To approximate the average behavior of ion channels with stochastic gating, a single-compartment, graded-potential Hodgkin-Huxley type model neuron was simulated. Channel “activity” (or open probability, “*U*” in equations 2-4) varied continuously between 0 and 1, whereas the “number of channels” (“*w”* in equations 2-4) was allowed to be any positive real number. The unit conductance of each channel was one.

For the plasticity rules, the constants were chosen to be:

Hebbian rule for layer 1 (equation 4) *θ* = -50 mV α = 0.1 β = 10^-7^

anti-Hebbian rule for layer 2 (equation 3) *θ* = -50 mV α = 1 β = 10^-7^

The null point *θ* was chosen to be midway between the K+ and non-selective cation reversal potentials. Besides the pattern of glutamate concentration, the plasticity rates were the only “free” parameters in the simulation (other parameters were chosen either to approximate known physical values or, in the case of channel subtypes, to span a spectrum of relevant kinetics or glutamate affinities). The greater value of α in the anti-Hebbian rule relative to the Hebbian rule was chosen to constrain growth in the number of glutamate-gated channels resulting from the positive feedback of the Hebbian rule (by making the negative feedback of the anti-Hebbian rule faster). The “passive” decay rate (β) was chosen to be much less than the “active” plasticity rate (α), which in turn was chosen to be slow in comparison to the rate of change in channel activities. Even slower plasticity rates might have been preferable. However, higher rates reduced the amount of computing time necessary for channel numbers to stabilize.

The membrane potential and channel activities were determined in the same manner as in the simulation of figure 2 described above. However, unlike that simulation, the glutamate-gated channels in this case had instantaneous kinetics. Because the selection of K+ channels by the anti-Hebbian rule (equation 3) was designed to be sensitive to temporal correlations in membrane voltage, assuming instantaneous kinetics in layer 1 and keeping the membrane time constant fixed at one time unit (rather than allowing it to vary with membrane conductance) simplified the interpretation of results.

The type 1 K+ channels were each gated by a single two-state sensor. There were four channel subtypes that differed in their kinetics, as shown in figure 3E (τ_Max_ = 10, 33, 100, or 333 time units). Other than their kinetics, they were identical to the K+ channels of figure 2 except that their half-maximal activation (V_1/2_) was –40 mV rather than –50 mV. The choice of –40 mV was not of fundamental importance, but it did better illustrate the model’s dynamics. This was because the lower activity of the K+ channels (Fig. 3E) caused membrane voltage in the early cycles to be almost constantly depolarized beyond –50 mV (Figure 3b). Because *θ* in the anti-Hebbian rule (equation 3) was –50 mV, K+ channels were added to the membrane until the average membrane voltage neared –50 mV. Membrane voltage therefore became gradually more hyperpolarized across cycles (Fig. 3B).

Type 2 K+ channels each had 2 layers of 4 sensors each. The sensors of layer 1 were not modeled realistically. Rather, they all “turned on” instantly whenever membrane voltage was depolarized beyond –25 mV. The sensors of layer 2 adapted to those of layer 1 with reversible first order kinetics, with time constants that varied across the five channel types as indicated in figure 3G (τ = 10, 33, 100, 333, or 1000 time units). The sensors of layer 1 reverted to the *off* state with irreversible first-order kinetics with a time constant in each channel type that was ten times the time constant of the layer 2 sensors of the same channel. A channel was open (conducting) only when all four sensors of layer 2 were *on* and at least one sensor of layer 1 was *on*. The open channel probability (or activity, *U*) (Fig. 3G) of a type 2 channel was:

(14)

where *u_1_* and *u_2_* were the activities of individual sensors in layers 1 and 2, respectively.

#### **RESULTS AND DISCUSSION**

**Plasticity Algorithms**

The term “*θ* “ refers to a voltage near the middle of the range that corresponds to the null point where the error is zero. Depolarization beyond *θ* would increase weights, whereas hyperpolarization would decrease weights. If the error is defined with respect to the predictions made by the sensors, then *θ* would not be a constant but would change over time. This can be seen by inspection of figure 2. Although the error is nearly zero at time points 1 and 3 of panel C, as shown by the probability distributions in panels E and G, the voltage at these two time points is quite different. One possibility is that there is machinery within the neuron that tracks the null point, by tracking recent membrane voltage, and modifies *θ* in the plasticity algorithms accordingly. Such a sliding threshold for plasticity has been proposed on other grounds (Sutton and Barto, 1981; Bienenstock et al., 1982). Alternatively, *θ* in the plasticity algorithms could simply be a constant voltage, in which case there would be a direct correspondence between membrane voltage and the functional error signal. The simulation illustrated in figure 3 set *θ* equal to –50 mV.

The plasticity algorithms (equations 3 and 4) do not require that a neuron’s inputs (synaptic and non-synaptic) be plastic in the usual sense, since the learning rate (α) could be so slow as to be negligible. When a neuron has experienced a similar pattern of inputs over a long period of time, it might be advantageous to reduce the learning rates. This could occur during development as input patterns become more familiar. Similarly, the stimulus patterns experienced by early sensory neurons may be so stereotyped over generations that their inputs have become “hard-wired” over evolutionary time scales. Even if a plasticity rule such as this is not being actively implemented within a neuron, it may have been implemented in the past. We might therefore be able to use the rule to explain which inputs the neuron selected, if we have knowledge of the history of the neuron’s inputs.

#### **Plasticity Mechanisms**

Relative to plasticity of inhibitory synapses and non-synaptic ion channels, plasticity at glutamate synapses is reasonably well understood (Malinow and Malenka, 2002). At a Hebbian glutamate synapse, the weight (*w*) of a particular synapse in equation 4 would typically be determined by the number of AMPA-type glutamate receptors, whereas the activity (*U*) would correspond to the number of glutamate-bound NMDA receptors. The joint voltage and glutamate dependence of the NMDA conductance would enable it to report the correlation between the two. The critical correlation should be between local synaptic glutamate concentration and the global, AMPA-mediated depolarization that may result after a brief delay (because of membrane capacitance and conduction delays). The higher glutamate affinity and slower kinetics of the NMDA receptor relative to the AMPA receptor could help to account for this brief delay. An excitatory synaptic input that is followed at short latency by a depolarization suggests to the neuron that that individual input contributed to the positive error and it should therefore be strengthened. An excitatory input coinciding with a negative error, or hyperpolarization, would be counteracting the error, and that synapse should be weakened. The temporal asymmetry introduced by the slower kinetics of the NMDA receptor (which might contribute to spike-timing dependent plasticity (e.g. Markram et al., 1997)), together with the hyperpolarizing influence of voltage-activated K+ channels, would insure that in the case of a barrage of synaptic inputs, the first synapses to become active would be strengthened most. Thus the neuron would learn to respond only to the earliest (and most predictive) in a stereotyped sequence of excitatory inputs. This feature of the present neuron model is similar to that previously proposed by Sutton and Barto (1981).

Little is known about either the rules or the mechanisms by which a neuron selects its non-synaptic ion channels. The plasticity algorithm proposed here (equation 3) would appear to require detection of the coincidence of a channel being open and the membrane being depolarized (or hyperpolarized). For example, a regulatory protein that is physically associated with a voltage-gated K+ channel could promote insertion of additional channels of the same type when activated by the combination of the open channel conformation and the high calcium that accompanies depolarization. However, a functional channel would not necessarily be required to drive plasticity. At glutamate synapses, NMDA-receptor mediated plasticity can occur at synapses which lack AMPA receptors and are therefore “silent.” It is advantageous to have independent mechanisms mediating plasticity (NMDA receptors) and synaptic weight (AMPA receptors). For example, it allows a synapse to have a weight near zero but to retain the potential to increase its weight in the case that activity patterns change in the future. An analogous independence could exist in the mechanisms that mediate induction and expression of plasticity in the case of non-synaptic channels. Just as the AMPA and NMDA receptors must be present at the same synapse, the voltage sensor that drives plasticity would need to share the same voltage-dependence and kinetic properties as the channel expressing the plasticity. The activity of this sensor would correspond to the activity *U* in equation 3, whereas the number of functional channels would correspond to *w*. The voltage sensors that regulate plasticity could be non-conducting channels of the same type as the conducting channels being regulated. Alternatively, the regulatory voltage sensor could be in a molecule distinct from the channel but which has matching voltage-dependent properties.

# Forms of Reward Feedback

Reward feedback is proposed to shape synaptic plasticity (equation 4). There are two critical aspects to this proposal. First, there is always a reward feedback signal present, even if it is provided only by natural selection over the course of generations. Second, the form and sophistication of the reward signal can vary greatly. Natural selection would obviously be a very slow and crude form of reward feedback. By contrast, selective attention in the neocortex could be the most sophisticated form of reward feedback. Shifts in attention, and corresponding changes in neuronal activity, can occur within hundreds of milliseconds or less. The cellular mechanisms of attention are not known. But in the context of the present theory, it would presumably involve the activity-dependent modulation of synaptic weights (equation 4) by top-down projections. This would require a large number of top-down projections, each of which would need to target a select group of synapses. Selective attention would therefore require the formation of precise circuitry, the development of which may itself require plasticity algorithms.

Another sort of reward signal could be provided by modulatory neurotransmitters. The best characterized example would be dopamine released by neurons with cell bodies located in the ventral midbrain (e.g. Montague et al., 1996; Schultz et al., 1997). Dopamine neurons are excited by events that are better than expected and suppressed by events that are worse then expected. This sort of reward signal would be appropriate for shaping the inputs to neurons that are close to the motor periphery, where inputs associated with good events should be strengthened, and those associated with bad events should be weakened. Indeed, dopamine has its effect primarily on neurons within or near to motor systems. However, the dopamine reward signal would appear to be inappropriate for neurons far from motor systems. For example, the stimuli of neurons in early visual and auditory systems should be selected to be informative about both good and bad events. Thus one can imagine that the appropriate reward signal to these neurons might be positive and strengthen synapses when events are either better or worse than expected (similar to the absolute value of the bidirectional dopamine signal). However, for some sensory modalities, the stimuli of neurons at very early stages of processing may have very little information about future reward (e.g. light intensity in a small region of space). In such cases, it may be advantageous not to have a dynamic reward feedback signal at all, since such a signal would generally fail to identify strongly reward-predictive stimuli. A simple error-maximizing (Hebbian) plasticity rule (equation 4 without a dynamic reward signal) should be sufficient to identify stimuli that are relatively unpredictable and thus provide the system with new information. Higher level neurons, which do receive a dynamic reward feedback signal, would then have the opportunity to link that information to future reward. For example, the orientation selectivity of simple cells in V1 could form through a Hebbian rule in the absence of dynamic reward feedback (Bienenstock et al., 1982). In contrast, a higher-level visual neuron in inferotemporal cortex might develop selectivity for faces or money, rather than other objects, under the influence of a reward feedback signal.

**The Temporal Predictions of Ion Channels**

A neuron’s predictions will be influenced by the channels that are available to it. A simple organism with a small genome might have a relatively small number of distinct channels, and it would therefore be highly constrained in the stimulus patterns that it could learn to recognize. By contrast, vertebrates are known to have numerous channel types, with the diversity of K+ channels being particularly striking (Hille, 2001). Here I consider three hypothetical types of channels and the stimulus patterns that they would be well suited to exploit.

The simplest ion channel would be gated by a single two-state sensor. Since its kinetics would be governed by a single exponential process, it would use the more recent past to predict the present and near future. In many cases this would yield quite accurate predictions, since the world tends not to change frequently. A high degree of ‘temporally local’ correlation in membrane voltage (such as that induced by slowly varying synaptic stimulation or by membrane capacitance) would favor voltage-regulated potassium channels with faster kinetics, since the most recent past would be the best predictor of the present. However, in the presence of high frequency variation in membrane voltage (which could, for example, be introduced within a small region of membrane by the stochastic opening of a small number of local ion channels), slower kinetics would be favored, since a better estimate can be made by averaging across a longer period of past voltages. It should be noted that the minimal amount of work required to open and close a channel imposes a limit on how rapidly a channel can be gated.

Although the present often matches the recent past, a simple exception to this rule would be a sinusoidal pattern. High intensity in the recent past could then be associated with low intensity in the near future. In this case, a K+ channel with appropriate inactivation kinetics might do better than a non-inactivating channel in predicting stimulus intensity, since it would activate on the rising phase of the wave but then inactivate “in anticipation” of the falling phase. It would therefore avoid the punishment that an anti-Hebbian rule would deliver to a non-inactivating K+ channel that remains open when the neuron is hyperpolarized near the trough of the wave. An alternative means of predicting and cancelling a sine-wave-like pattern of excitatory synaptic input could be through anti-Hebbian-selected, depolarization-activated sodium or calcium channels (although some of these channels are presumed to have distinct and specialized roles in long-distance communication and the regulation of plasticity, respectively). The activation of such conductances, induced during the depolarized phase, could occur with slow enough kinetics that the channels would not become substantially activated until the excitatory input had declined on the falling phase of the cyclic pattern. The voltage-gated conductance would thus be out of phase with the excitatory synaptic input, and the two would counterbalance one another. The sum of the two depolarizing conductances (inward currents) would therefore be roughly constant, and could be effectively cancelled by a non-inactivating K+ conductance (outward current).

A more complex temporal pattern could consist of two events, in which a second event of high stimulus intensity consistently follows a first event by an interval of arbitrary length (Fig. 3A). Such a pattern may not be prominent at early sensory levels, but it is presumably important at later, more “cognitive” stages of processing. One way in which this could be accomplished is with a two-layer K+ channel such as the “type 2” channels of figure 3. For example, a large membrane depolarization could trigger a rapid but long-lasting phosphorylation of sensors in the first layer. This would act as a “time-stamp” that would begin a slower, voltage-independent activation of sensors in the second layer. The channel would open only when all sensors of the second layer are activated. The temporal prediction associated with the channel’s conductance would depend on both the kinetics of its individual sensors as well as their stoichiometry (Fig. 3G). The anti-Hebbian algorithm (equation 3) selects this type of channel if its kinetics are appropriate to the interval between the two events (Fig. 3H). Such a channel may or may not exist. The important point is that the known complexity of channel gating, and its regulation by associated proteins, provides a rich substrate for making at least moderately sophisticated predictions of future stimulus intensity based upon prior temporal information held within channels themselves.

**Action Potentials and Presynaptic Terminals**

The discussion has thus far focused entirely on membrane voltage in a single compartment neuron that lacks action potentials. Although many neurons have action potentials, neurons that do not need to transmit their output over long distances often do not. Thus a “digitized,” action-potential output may be specialized for reliable communication over longer distances (in axons or dendrites), although this would come at the cost of a relatively crude representation of the “analog” information in the membrane voltage (de Ruyter van Steveninck and Laughlin, 1996; Juusola and French, 1997). In the present model, an action potential would presumably be triggered when the voltage is depolarized past a threshold that lies just beyond the midpoint of the equilibrium potentials of layers 1 and 2 (~–50 mV, or *θ* in equations 3 and 4). An action potential would therefore signal a positive prediction error.

A neuron’s pre-synaptic terminals receive the output of its somatodendritic compartment, and “decide” whether or not to release neurotransmitter. It is proposed that information in a synaptic terminal predicts the output of the somatodendritic compartment, and that a vesicle of neurotransmitter is released only when this excitatory input exceeds expectations. Thus the function of a synaptic terminal would be analogous to the function of the somatodendritic compartment. It may seem redundant to have two separate compartments acting in series to predict the same stimulus. However, the two compartments may be far apart from one another, and it would be more efficient for other neurons to provide information to the closer of the two.

Because the presynaptic terminal is proposed to function as a discrete neural element, functionally analogous to a somatodendritic compartment, modulation of pre-synaptic function *would not* contribute directly to the changes in synaptic weights described in equations 3 and 4, which would be determined solely by postsynaptic factors. Suppression of vesicle release would occur when the excitatory axonal input to the terminal at a given moment is predictable, or when feedback from higher neurons, possibly including the postsynaptic neuron, indicates that the axonal input is not relevant to future reward.

# SUPPORTING REFERENCES

Bienenstock EL, Cooper LN, Munro PW (1982) Theory for the development of neuron selectivity: orientation and binocular specificity in visual cortex. J Neurosci 2: 32-48.

Boeker EA (1984) Simple integrated rate equations for reversible bimolecular reactions. Experientia 40: 453-456.

Dayan P, Abbott LF (2001) Theoretical Neuroscience. Cambridge, MA: MIT Press.

de Ruyter van Steveninck RR, Laughlin SB (1996) The rate of information transfer at graded potential synapses. Nature 379: 642-645.

Feller W (1950) An Introduction to Probability Theory and its Applications. New York: Wiley.

Hille B (2001) Ionic Channels of Excitable Membranes. Sunderland, MA: Sinauer.

Jaynes ET (2003) Probability Theory: The Logic of Science. Cambridge, England: Cambridge University Press.

Juusola M, French AS (1997). The efficiency of sensory information coding by mechanoreceptor neurons. Neuron 18: 959-968.

Keynes RD, Rojas E (1974) Kinetics and steady-state properties of the charged system controlling sodium conductance in the squid giant axon. J Physiol 239: 393-434

Knill DC, Richards RW (Editors) (1996) Perception as Bayesian Inference*.* Cambridge, England, Cambridge University Press.

Kording KP, Wolpert DM (2004) Bayesian integration in sensorimotor learning. Nature 427: 244–247.

Laplace PS (1819) Essai Philosophique sur les Probabilités. Paris: Courcier Imprimeur.

Malinow R, Malenka RC (2002) AMPA receptor trafficking and synaptic plasticity. Ann Rev Neurosci 25: 103–126.

Markram H, Lubke J, Frotscher M, Sakmann B (1997) Regulation of synaptic efficacy by coincidence of postsynaptic APs and EPSPs. Science 275: 213-215.

Montague PR, Dayan P, Sejnowski TJ (1996) A framework for mesencephalic dopamine systems based on predictive Hebbian learning. J Neurosci 16: 1936-1947.

Niemeier M, Crawford JD, Tweed D (2003) Optimal transsaccadic integration explains distorted spatial perception. Nature 422: 76-80.

Purves D, Lotto RB (2003) Why We See What We Do: An Empirical Theory of Vision. Sunderland MA: Sinauer Assoc.

Rao RPN, Olshausen BA, Lewicki, MS (Editors) (2002) Probabilistic models of the brain: perception and neural function. Cambridge, MA: MIT Press.

Rieke F, Warland D, de Ruyter van Steveninck RR, Bialek W (1997) Spikes: Exploring the Neural Code. Cambridge, MA,: MIT Press.

Schultz W, Dayan P, Montague PR (1997) A neural substrate of prediction and reward. Science 275: 1593-1599.

Seidenberg MS (1997) Language acquisition and use: learning and applying probabilistic constraints. Science 275: 1599–1603.

Shannon CE (1948) The mathematical theory of communication. Bell Syst Tech J 27: 379-423.

Singh K, Scott SH (2003) A motor learning strategy reflects neural circuitry for limb control. Nat Neurosci 6: 399-403.

Sutton RS, Barto, AG (1981) Toward a modern theory of adaptive networks: expectation and prediction. Psychol Rev 88: 135–170.

von Helmholz H (1896) Concerning the perceptions in general. In: Treatise on Physiological Optics. Reprinted in Visual Perception, Yantis S, editor. Philadelphia: Psychology Press, 2001, pp. 24 - 44.

Weiss Y, Simoncelli EP, Adelson EH (2002) Motion illusions as optimal percepts. Nat Neurosci 5: 598-604.

Yang Z. Purves D (2003) A statistical explanation of visual space. Nat Neurosci 6: 632-640.
